# Mechanisms of Resistance to Small Molecules in Acute Myeloid Leukemia

**DOI:** 10.3390/cancers15184573

**Published:** 2023-09-15

**Authors:** Tonio Johannes Lukas Lang, Frederik Damm, Lars Bullinger, Mareike Frick

**Affiliations:** 1 Department of Hematology, Oncology and Cancer Immunology, Charité–Universitätsmedizin Berlin, Corporate Member of Freie Universität Berlin and Humboldt Universität zu Berlin, 13353 Berlin, Germany; 2 German Cancer Consortium (DKTK) and German Cancer Research Center (DKFZ), 69120 Heidelberg, Germany

**Keywords:** AML, small molecule, gene mutations, resistance, FLT3, IDH1/IDH2, BCL2, venetoclax, targeted therapy, precision medicine

## Abstract

**Simple Summary:**

Acute myeloid leukemia (AML) is a dangerous cancer of the blood. In recent years, a series of drugs was approved to specifically target misdirected processes in the cancerous cells. These so-called “small molecules” substantially improved therapeutic outcomes, but eventually leukemia returns in most patients. In this review, we summarize the current state of knowledge regarding the mechanisms that lead to failure of the most frequently used new therapies and introduce potential strategies to overcome the mechanisms associated with disease recurrence.

**Abstract:**

In recent years, great progress has been made in the therapy of AML by targeting cellular processes associated with specific molecular features of the disease. Various small molecules inhibiting FLT3, IDH1/IDH2, and BCL2 have already gained approval from the respective authorities and are essential parts of personalized therapeutic regimens in modern therapy of AML. Unfortunately, primary and secondary resistance to these inhibitors is a frequent problem. Here, we comprehensively review the current state of knowledge regarding molecular processes involved in primary and secondary resistance to these agents, covering both genetic and nongenetic mechanisms. In addition, we introduce concepts and strategies for how these resistance mechanisms might be overcome.

## 1. Introduction

AML is an aggressive blood cancer characterized by uncontrolled proliferation of malignant hematopoietic stem and progenitor cells. Comprehensive genomic studies have revealed the genetic complexity of this disease, leading to refined classification systems and risk stratification [1,2,3,4]. 

For a long time, therapy of AML has been purely chemotherapy-based, with cytarabine and anthracyclines being the standard of care in most first-line settings [5,6]. However, with increasing knowledge of the genetic heterogeneity of this disease and associated efforts to develop agents that specifically target genetic lesions, the therapeutic landscape has started to change. In 2017, the FLT3 inhibitor midostaurin was the first small molecule in the AML setting that gained approval from the authorities [7]. Since then, a multitude of small molecules have been developed. These inhibitors have already been approved or are currently at different stages of preclinical or clinical testing. Despite the unquestioned clinical success of these agents, primary resistance or relapses are an immense clinical problem. Understanding and overcoming resistance mechanisms is, therefore, a major challenge for clinicians and researchers. 

In this review, we focus on the current knowledge of genetic and nongenetic mechanisms of resistance of the clinically most relevant, authority-approved small molecules in the context AML. These comprise various FLT3 inhibitors, inhibitors of IDH1/IDH2, and a BCL2 inhibitor. To this end, PUBMED was searched for the terms listed in the keywords section and names of individual inhibitors (e.g., ivosidenib), and the literature was critically reviewed. The review presented here focuses on articles providing clinical or mechanistic reports on resistance to small molecules in AML therapy. 

## 2. FLT3 Inhibitors

FMS-like tyrosine kinase 3 (FLT3) is a protein encoded by the *FLT3* gene located on chromosome 13q12. It is primarily expressed in hematopoietic progenitor cells [8] and is one of the most frequently mutated genes in AML [9]. FLT3 consists of five extracellular immunoglobulin-like domains, a transmembrane sequence, a juxtamembrane region, and two interrupted kinase domains. The binding of the FLT3 ligand triggers a conformational change, leading to homodimerization, autophosphorylation steps, and the activation of its intrinsic tyrosine kinase function [10]. This activation promotes cell proliferation and inhibits apoptosis via the PI3K (phosphatidylinositol 3-kinase), STAT3 (signal transducer and activator of transcription 3), and RAS/MAPK (mitogen-activated protein kinase) signaling pathways.

Roughly one-third of patients with AML have activating *FLT3* mutations [11]. In approximately two-thirds of cases, the mutations are in-frame internal tandem duplications (ITDs), and in one-third of cases, mutations are missense point mutations within the tyrosine kinase domain (TKD), frequently encoding for D835 or I836 [12]. Patients with AML with *FLT3* mutations often exhibit high leukocyte and blast counts in both peripheral blood and bone marrow [13].

In contrast to the previous version of the European LeukemiaNet (ELN) risk classification of AML, the most recent version categorizes all *FLT3*-ITD mutations in the intermediate risk group. This reclassification is primarily due to the fact that *FLT3* mutations have become a “druggable” target with the development of FLT3 inhibitors, which nicely demonstrates that targeted therapy can impact disease outcome and the prognostic value of genomic aberrations [2]. 

### 2.1. Overview of FLT3 Inhibitors

In recent years, several small-molecule tyrosine kinase inhibitors (TKIs) targeting the ATP-binding site of the FLT3 kinase or adjacent structures have been developed [14,15]. First-generation FLT3 inhibitors possess multikinase activity and, hence, target other kinases like KIT, PDGFR, VEGFR, RAS/RAF, and JAK2 kinases to various degrees. Sorafenib and midostaurin both belong to this group [16]. Sorafenib was shown to reduce the risk of AML relapse when used as maintenance therapy after hematopoietic stem cell transplantation for *FLT3*-ITD-positive AML in the SORMAIN study [17]. In addition, it has also improved progression-free survival (PFS) and event-free survival (EFS) when used in combination with chemotherapy in newly diagnosed AML in the SORAML study [18,19]. While sorafenib is not approved for the treatment of AML, midostaurin is the first FDA- and EMA-approved first-generation FLT3 inhibitor that has shown efficacy in prolonging overall survival (OS), EFS [7], and reduction in relapse rates [20] in *FLT3*-mutated AML within the large, randomized phase-III RATIFY trial. 

Second-generation FLT3 inhibitors more specifically bind to FLT3 than first-generation FLT3 inhibitors and, hence, have shown improved efficacy. They include quizartinib, gilteritinib, and crenolanib [16]. Quizartinib demonstrated an OS benefit in the treatment of relapsed patients with AML with *FLT3*-ITD mutations compared to standard salvage chemotherapy in the QUANTUM-R trial [21]. Lately, efficacy of quizartinib was also shown in a first-line setting when used as an alternative to midostaurin in addition to standard chemotherapy in *FLT3*-ITD-mutated AML within the QUANTUM-First study [22]. Gilteritinib, another second-generation FLT3 inhibitor, has been established as standard therapy for the treatment of relapsed or refractory (r/r) *FLT3*-mutated AML. It gained approval from the FDA and EMA based on the data from the ADMIRAL study [23]. 

FLT3 inhibitors can also be classified into type-1 and type-2 inhibitors. While type-1 inhibitors bind to the active conformation of FLT3, type-2 inhibitors bind to its inactive conformation. Pharmacologically, type-1 inhibitors can target both ITD and TKD mutations, while type-2 inhibitors do not effectively inhibit *FLT3*-TKD-mutated disease. Midostaurin, gilteritinib, and crenolanib belong to the group of type-1 inhibitors, while sorafenib and quizartinib are classified as type-2 inhibitors [16,24]. 

### 2.2. Mechanisms of Resistance to FLT3 Inhibition

Despite promising results, response durations in patients treated with FLT3 inhibitors remain relatively short when used as monotherapy in the r/r setting (4–14 months) [21,23]. Therefore, understanding the mechanisms of primary (refractory disease) and secondary (relapsed disease) resistance is of great clinical relevance. 

#### 2.2.1. Genetic Mechanisms Causing Resistance

**Figure 1:**
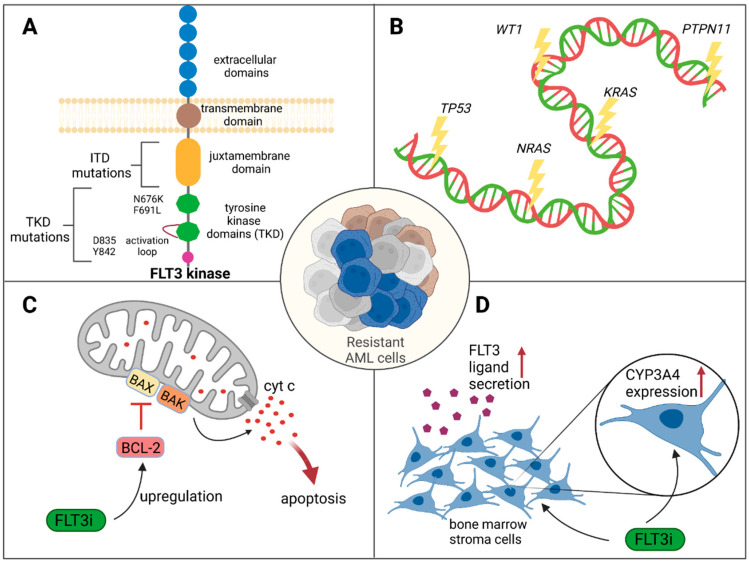
Mechanisms of resistance to FLT3 inhibition. (**A**): Scheme of FLT3 kinase. Mutations of particular relevance for resistance are marked. (**B**): Mutations shown to confer resistance to FLT3 inhibition. (**C**): FLT3 inhibition leads to upregulation of antiapoptotic BCL2, leading to decreased apoptosis. (**D**): Upon treatment with FLT3 inhibitors, bone marrow stromal cells can secrete increased amounts of FLT3 ligands. In addition, stromal cells can upregulate CYP3A4, leading to rapid degradation of the inhibitor. FLT3i: FLT3 inhibitor. Created with BioRender.com.

On-target mutations (Figure 1A): The acquisition of on-target or secondary *FLT3* mutations has been identified as a mechanism of secondary resistance to treatment with FLT3 inhibitors. *FLT3* F691L, a well-known gatekeeper mutation within the active site of the TKD, confers resistance to all clinically used FLT3 inhibitors [25,26,27,28]. Other important mutations in this context are *FLT3* N676K in TKD, which was shown to confer resistance to midostaurin [29], and K429E, which confers resistance to crenolanib [30]. Mutations in the activation loop of FLT3, such as *FLT3* D835 (D835F/V/Y) or *FLT3* Y842C/H, only confer resistance to type-2 FLT3 inhibitors like quizartinib [28] or sorafenib [31]. The emergence of secondary *FLT3* mutations is also considered one of the main factors contributing to the drop in composite CR (CRc) rates during sequential exposure to FLT3 inhibitors [32]. However, a comprehensive analysis by Schmalbrock et al. investigating genetic causes for midostaurin resistance in *FLT3*-ITD-mutated AML demonstrated that secondary *FLT3*-ITD mutations occurred in only 11% of patients at the time of r/r disease. Instead, the authors observed an outgrowth of clones that lost the *FLT3*-ITD mutation and clones harboring signaling pathway mutations downstream of FLT3 as the most prevalent mechanisms of resistance in their patient cohort [26]. 

Activation of alternative signaling pathways by off-target mutations (Figure 1B): Various studies using comprehensive, next-generation sequencing (NGS) approaches have shown that the activation of alternative signaling pathways, like PI3K/AKT/mTOR [33], RAS/RAF/MEK/ERK [26,34,35], JAK/STAT [34,36], and SRC family kinases [37], by off-target mutations is an important mechanism of resistance to FLT3 inhibition. A study conducted by Alotaibi et al. investigated pretreatment bone marrow samples (primary resistance cohort) and pre- and posttreatment bone marrow samples (secondary resistance cohort) of a large patient cohort receiving FLT3 inhibitors. Within the secondary resistance cohort, they identified off-target mutations in epigenetic modifiers (16%), *RAS*/*MAPK* pathway genes (13%), *WT1* (7%), and *TP53* (7%). Mutations in genes of the RAS/MAPK pathway were commonly observed as mechanisms of resistance to type-1 FLT3 inhibitors (29%). Nonresponders to FLT3 inhibition had a higher variant allele frequency (VAF) of *RAS* mutations (31% in nonresponders vs. 6% in responders; *p* = 0.19) as identified NGS, likewise indicating a role of the RAS pathway in this context [34]. On a similar note, the study by Schmalbrock et al. using whole-exome sequencing (WES) of samples from patients with AML undergoing midostaurin treatment revealed enrichment of several mutated genes at disease progression, including *WT1*, *NRAS*, *KRAS*, and *IDH1*. In addition, the authors observed acquired mutations in genes associated with chromatin cohesin/splicing (*ASXL1*, *U2AF1*, *ZBTB7A*, and *SF3B1*) upon resistance development in these patients, indicating a role for associated processes in the development of resistance [26]. Similarly, mutations in *NRAS*, *PTPN11*, *ABL1*, *BCORL1*, *CEBPA*, *WT1*, and *IDH1* emerged under treatment with crenolanib in another study, leading to resistance, as shown by WES [30]. In a study by McMahon et al., targeted sequencing revealed that 15 out of 41 patients treated with gilteritinib exhibited mutations within the RAS/MAPK pathway as a mechanism of secondary resistance. Additional single-cell analyses revealed complex clonal selection and evolution processes involving both on-target and off-target mutations, pointing to the highly heterogeneous process of developing resistance [35].

#### 2.2.2. Nongenetic Mechanisms Causing Resistance

Over-expression of antiapoptotic proteins (Figure 1C): The overexpression of antiapoptotic proteins is a critical mechanism of resistance to FLT3 inhibitors. AML cells can overcome FLT3 inhibition by upregulating antiapoptotic proteins [38]. In cell line models, the overexpression of BCL2 (B-cell lymphoma 2) family proteins was found to confer resistance to FLT3 inhibitors, enabling hematopoietic cells to evade apoptosis [39]. Notably, the effect of this overexpression can be counteracted using BCL2 inhibitors. Thus, the upregulation of BCL2 by FLT3 inhibition presents a potential therapeutic target, which is discussed in detail in Section 2.3 and Section 4.3. Moreover, high expression levels of P-glycoprotein efflux pumps can reduce the levels of apoptosis induced by FLT3 inhibition [40].

Role of microenvironment/stem cell niche (Figure 1D): The bone marrow microenvironment, known as the niche, plays a crucial role in facilitating the growth, survival, and development of drug resistance in leukemic (stem) cells [41,42]. Within this niche, bone marrow stroma cells secrete the FLT3 ligand, a naturally occurring growth factor that has been identified as a mediator of resistance to FLT3 inhibition. Sato et al. observed higher levels of FLT3 ligands in relapsed than in newly diagnosed patients with AML. They also demonstrated that FLT3 ligands mitigated the effects of FLT3 inhibition and cytotoxicity in vitro [43]. Additionally, Chang et al. showed that primary stromal cells within the bone marrow niche could promote the degradation of FLT3 inhibitors, such as sorafenib, quizartinib, and gilteritinib, through the expression of CYP3A4. This enzymatic degradation led to reduced activity of the inhibitors in vitro. Interestingly, the effect could be reversed by clarithromycin, a potent CYP3A4 inhibitor [44]. Lastly, cytokines like GM-CSF and TPO secreted by bone marrow stromal cells, as well as hypoxia via HIF-1α signaling, can confer FLT3 inhibitor resistance via AXL upregulation in vitro [45,46]. Moreover, FLT3 inhibitor efficacy can be diminished by increased binding of the inhibitors to plasma proteins [47]. 

### 2.3. Overcoming Resistance to FLT3 Inhibitors

Several approaches exist to overcome resistance to FLT3 inhibition. These include the development of next-generation inhibitors, combination therapies with cytotoxic chemotherapy or other targeted agents, targeting the microenvironment, and utilizing dual-targeted inhibitors.

A series of next-generation FLT3 inhibitors are currently at different stages of preclinical and clinical investigation. Among these, sitravatinib was more effective than gilteritinib in xenograft models derived from patient blasts carrying *FLT3*-ITD mutations. Of note, the predicted binding sites of sitravatinib do not include the F691L residue [48]. The covalently binding, irreversible FLT3 inhibitor FF-10101 exhibited high efficacy in AML cell lines harboring mutations at the D835, Y842, and F691 residues of the FLT3 kinase domain [49]. It also demonstrated clinical activity in FLT3 inhibitor refractory patients in a phase-I trial [50]. 

Another strategy to overcome resistance to FLT3 inhibition is the addition of the BCL2 inhibitor venetoclax to induce apoptosis. A multitude of studies have demonstrated the synergistic nature of this approach in the preclinical setting [51,52,53,54]. A recent phase-1b study enrolling 61 patients combing venetoclax and gilteritinib led to high modified CR rates (mCRC; 75% in FLT3-mutated patients) and *FLT3* molecular response rates, even in patients with prior FLT3 inhibitor treatment [55]. Interestingly, a drug-screening approach to primary AML cells also identified gilteritinib and venetoclax as a synergistic drug combination for *FLT3* wild-type high-risk AML. Mechanistically, the combination led to downregulation of antiapoptotic protein myeloid leukemia 1 (MCL1) via altered signaling of the involved ERK pathway [56]. Combinations of FLT3 inhibitors with other targeted agents have also been tested in various settings. Inhibitors targeting the JAK/STAT5 or PI3K/mTOR pathways [57,58,59], as well as FGFR1 [60] or CXCR4 [61] inhibition in the microenvironment, have shown the potential to act synergistically with FLT3 inhibitors. Preclinical studies have indicated that inhibiting autophagy via BTK inhibition or using the novel FLT3/BTK/aurora kinase inhibitor luxeptinib can overcome FLT3 inhibitor resistance [62]. 

## 3. IDH Inhibitors

The isocytrate dehydrogenase (IDH) enzymes catalyze the oxidative decarboxylation of isocitrate to α-ketoglutarate (α-KG). Recurrent mutations in the iso-enzymes IDH1 and IDH2 occur in approximately 20% of de novo AML [9]. These mutations mainly affect R132 in *IDH1* and R140 and R172 in *IDH2* [63,64,65]. These mutations alter the biochemical function of the enzymes, as they lead to a reduction in α-KG to the oncometabolite 2-hydroxyglutarate (2-HG) [66]. 2-HG competitively inhibits α-KG-dependent enzymes [67], including the ten-eleven translocation (TET) family of 5-methylcytosine hydroxylases, thereby interfering with epigenetic processes. Finally, these processes impair hematopoietic differentiation and promote malignant transformation [68,69]. Inhibition of IDH1 or IDH2 in the presence of an oncogenic mutation, therefore, suppresses the production of the oncometabolite 2-HG and induces hematopoietic differentiation, making pharmacologic IDH inhibition a highly attractive therapeutic approach [70,71].

### 3.1. Overview of IDH Inhibitors

*IDH1*-mutated AML can be effectively treated with the orally available IDH1 inhibitor ivosidenib. Effectiveness as monotherapy in newly diagnosed AML with *IDH1* mutation was demonstrated in patients ineligible for intensive chemotherapy [72]. Effectiveness of IDH1 inhibition with ivosidenib in r/r *IDH1*-mutated AML was likewise shown [73]. Encouraging results were also seen when combining ivosidenib with azacytidine in patients with newly diagnosed *IDH1*-mutated AML ineligible for intensive chemotherapy in the phase-III AGILE trial. Here, the combination of ivosidenib with azacytidine led to significantly improved EFS and OS [74]. Based on these studies, ivosidenib gained FDA approval as a monotherapy for patients with *IDH1* mutation in r/r AML or elderly patients ≥75 years or not suitable for intensive induction chemotherapy in 2018 and 2019, respectively. In 2022, ivosidenib was approved as a first-line therapy in combination with azacytidine for patients with *IDH1* R132 mutation not eligible for intensive induction chemotherapy. EMA approval for this combination therapy followed in May 2023. 

Olutasidenib is another orally available FDA-approved IDH1 inhibitor. In r/r patients with AML, olutasidenib led to overall response rates of almost 50% and a median duration of response of 25.9 months in those patients achieving CR/CRh (CR with partial hematologic recovery) [75], which is remarkably longer than the reported 8.2 months for patients achieving CR/CRh with ivosidenib [73]. Olutasidenib was also evaluated in combination with azacytidine in r/r AML and de novo AML. In these settings, CR/CRh rates of 15% in patients with r/r AML and 54% in patients with newly diagnosed AML were achieved. OS rates were 12.1 months and not reached, respectively [76].

Enasidenib is a first-in-class orally available inhibitor of mutant *IDH2* that was granted FDA approval for r/r AML in 2017. In a pivotal phase-I/II study, enasidenib led to overall response rates of approximately 40% in patients with *IDH2*-mutated AML. Of note, while median OS was 9.3 months for the total cohort, patients attaining CR (19.3%) had a median OS of 19.7 months [77]. In a study investigating enasidenib in elderly patients with newly diagnosed AML with *IDH2* mutation, overall response rates of more than 30% were achieved [78]. Likewise, in de novo AML with *IDH2* mutation, the combination of enasidenib and azacytidine led to a significantly improved overall response when compared to azacytidine alone [79]. 

### 3.2. Mechanisms of Resistance to IDH Inhibition

Mechanisms of primary or secondary resistance to IDH inhibitors are manifold and comprise genetic, as well as nongenetic, mechanisms. 

#### 3.2.1. Genetic Mechanisms Causing Resistance

VAF/clone size: It is conceivable that the clone size can affect response and resistance patterns to targeted therapy. However, according to a study by Choe et al., there was no correlation between mutant *IDH1* clone size and achievement of CR following ivosidenib monotherapy [80]. Similar observations were made by Amatangelo et al., who reported no correlation between mutant *IDH2* clone size and response to single-agent enasidenib therapy [81]. 

**Figure 2:**
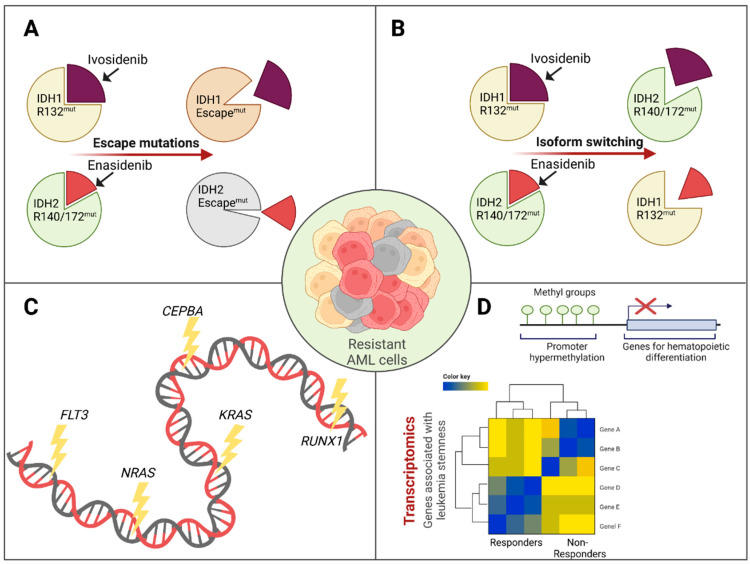
Mechanisms of resistance to IDH inhibition. (**A**): Escape mutations in the respective enzyme lead to resistance. (**B**): Isoform switching causes resistance to IDH inhibitors. (**C**): Mutations associated with primary or secondary resistance. (**D**): Methylomics and transcriptomics revealing leukemia stemness as additional factor for resistance to IDH inhibition. Created with BioRender.com.

Escape mutations in IDH enzymes/second site mutations (Figure 2A): Escape mutations that are likely to restore the pathologic metabolic capabilities of IDH enzymes have been described for both *IDH1*- and *IDH2*-mutated AML treated with ivosidenib and enasidenib, respectively. In a study by Choe et al. investigating mechanisms of resistance in 179 patients treated with ivosidenib, 20 second-site mutations in *IDH1* were detected at relapse or progression that either affected the binding pocket of ivosidenib or its cofactor NADPH or that led to hypothesized structural changes, preventing the interaction between ivosidenib and *IDH1* [80]. Likewise, second-site mutations leading to secondary resistance to enasidenib have been described for *IDH2* mutant AML. In specific, Intlekofer et al. describe two cases with newly acquired mutations on the second *IDH2* allele, which led to restoration of 2-HG production and AML relapse [82].

Isoform switching (Figure 2B): Another interesting mechanism of resistance to IDH inhibition is the restoration of 2-HG production by isoform switching, i.e., acquisition of *IDH2* mutations in the context of IDH1 inhibition and vice versa. This mechanism was described by Harding et al. in a series of two patients with AML and *IDH1* mutation receiving ivosidenib. After initial response to the therapy, both patients suffered from relapse that could be attributed to the outgrowth of *IDH2* R140Q-mutated clones [83]. The same mechanism was also described by Choe et al. [80]. Likewise, Wang et al. observed isotype switching in one case with initially *IDH2*-mutated AML treated with enasidenib that developed a de novo *IDH1* mutation at relapse [84]. 

Co-occurring mutations (Figure 2C): Co-occurring mutations are an important mechanism of resistance in both primary and secondary resistance. Various studies have consistently described mutations in receptor tyrosine kinase (RTK) pathways to be frequently associated with resistance and/or relapse. Here, mutations in *RAS* genes [80,81,85] and *FLT3* [80,84,85,86] are of particular relevance. In a study by Wang et al., clonal architecture at relapse was investigated via single-cell analysis. In the analyzed *IDH2* mutant patient who had received enasidenib, leukemic clones harboring *NRAS* and *KRAS* mutations arose independently from the *IDH2* mutant clone, highlighting clonal selection processes [84]. Likewise, mutations in the hematopoietic transcription factors *RUNX1* and *CEBPA* have been found in patients with both primary or secondary treatment failure of IDH inhibition [80,84]. This is most likely due to the fact that these mutations interfere with the myeloid differentiation induced by pharmacologic 2-HG suppression [84]. Two studies have shown that a lower number of co-occurring mutations is significantly associated with response to enasidenib in *IDH2*-mutated AML [81,85].

#### 3.2.2. Nongenetic Mechanisms Causing Resistance

Levels of 2-HG: Contradictory data exist for the question of whether the suppression levels of 2-HG correlate with response or resistance to IDH inhibition. In a study by Stein et al., patients with *IDH2* R172 mutation showed a significantly stronger reduction in 2-HG levels than nonresponders. However, in patients with *IDH2* R140 mutation, 2-HG levels were effectively suppressed, irrespective of response category [85]. On the contrary, Amatangelo et al. reported strong 2-HG suppression that did not correlate with response for both mutations [81]. While second-site mutations and isoform switching are effective escape mechanisms to restore 2-HG production and subsequent relapse in some patients, Quek et al. could show that most relapses occurred despite effective and ongoing 2-HG suppression. This implied that 2-HG independent mechanisms frequently caused relapse and resistance [86]. 

Leukemia stemness (Figure 2D): In the study by Wang et al. investigating alternative mechanisms of resistance to ivosidenib or enasidenib, comprehensive methylation and transcriptional data were generated for 60 patients. Interestingly, they described hypermethylation of various promotors associated with hematopoietic differentiation in various pretreatment samples with subsequent poor response to IDH inhibition. In line with these findings, they also reported transcriptional profiles associated with leukemia stemness associated with primary resistance to IDH inhibition [84]. 

### 3.3. Overcoming Resistance

Although responses to IDH inhibition are often long-lasting, relapses eventually occur. As outlined above, clonal heterogeneity and outgrowth of resistant clones seem to be major sources of resistance. Mutations that confer resistance often affect pathways not directly related to IDH inhibition (e.g., mutations of the RAS pathway), highlighting the necessity of hitting multiple targets using combination therapies. With respect to IDH inhibitors, multidrug combination regimens with HMAs and BCL-2 inhibitors yield promising results and are detailed in Section 4.3.

## 4. BCL2 Inhibitors

BCL2 and the related proteins BCLXL and MCL1 are antiapoptotic proteins that prevent apoptosis by stabilizing the outer mitochondrial membrane, thus circumventing permeabilization and cytochrome c release [87]. BCL2 is overexpressed in 80–90% of AML cases, leading to disturbed apoptosis and making BCL2 an attractive therapeutic target [88,89]. 

### 4.1. Pharmacologic Inhibition of BCL2

Venetoclax is an orally available BH3 mimetic that inhibits BCL2 and, hence, induces apoptosis via release of the proapoptotic proteins BAK and BAX. In patients with AML, it has limited efficacy when used as a monotherapy in r/r patients [90]. However, when combined with hypomethylating agents (HMAs) or low-dose cytarabine, response rates markedly increase, as shown in the phase-III VIALE-A and VIALE-C trials [91,92]. In the VIALE-A trial, combining azacytidine with venetoclax led to significantly improved CR rates and longer OS as compared to azacytidine monotherapy [91]. These results led to approval of venetoclax in these combinations by the FDA and EMA (EMA only approved combination therapy with HMAs). The effectiveness of the combination of azacytidine and venetoclax seems to be at least partly due to the fact that azacytidine induces the proapoptotic protein NOXA, priming AML cells for venetoclax-mediated apoptosis [93]. 

### 4.2. Mechanisms of Resistance

#### 4.2.1. Genetics of Response and Resistance to Venetoclax

The mutational pattern of AML plays an important role in response and resistance to venetoclax and can be used to predict response to therapy and relapse risk. A relevant number of mutations are associated with favorable outcomes in this setting and are, therefore, briefly introduced here as well.

**Figure 3:**
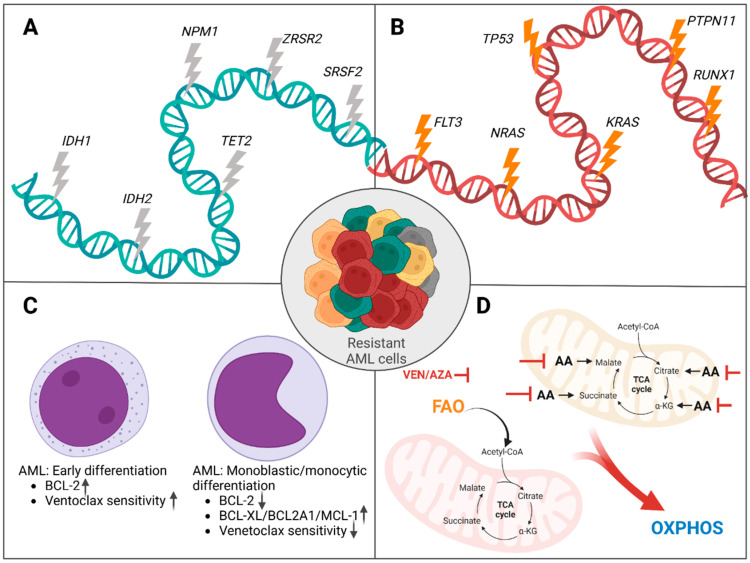
Mechanisms of response/resistance to venetoclax. (**A**): Mutations associated with favorable response to venetoclax. (**B**): Mutations associated with resistance to venetoclax. (**C**): Monocytic differentiation is associated with resistance to venetoclax. Monocytic differentiation of AML blasts is associated with downregulation of BCL2, whereas other proapoptotic proteins are upregulated. (**D**): Leukemic cells rely on amino acids for metabolization in the TCA cycle and subsequent OXPHOS. Venetoclax/azacytidine inhibits amino acid processing. Switching to fatty acid oxidation is a potential mechanism of resistance. VEN/AZA: venetoclax/azacytidine; AA: amino acid; FAO: fatty acid oxidation; OXPHOS: oxidative phosphorylation. Created with BioRender.com.

Mutations associated with response (Figure 3A): A multitude of studies have shown that the presence of mutations in *IDH1* and *IDH2* is associated with a favorable response pattern in monotherapy [90,94], as well as in combination therapy with azacytidine or decitabine [95,96], low-dose cytarabine [92], or intensive chemotherapy [97]. An in vitro study showed that 2-HG produced by *IDH1*/*IDH2*-mutated cells suppressed cytochrome c oxidase (COX) and, thus, lowered the threshold of mitochondria to initiate apoptosis via BCL2 inhibition. Therefore, *IDH1*/*IDH2* mutations and BCL2 inhibition are an example of synthetic lethality [98]. Due to the biochemical link between the IDH enzymes and epigenetic modifications catalyzed by TET enzymes, it is not surprising that mutations in *TET2* are likewise associated with favorable response to venetoclax-based therapeutic regimens [99]. Likewise, in studies involving venetoclax, *NPM1* mutations have been associated with favorable outcome parameters such as higher CR rates, higher blast reductions, or increased OS [91,92,100,101,102]. However, the molecular mechanisms behind the therapeutic response remain unclear [103]. In addition, mutations in the splicing factor genes *SRSF2* and *ZRSR2* have been reported to be associated with beneficial outcomes in studies involving venetoclax in AML treatment [94,101,102]. 

Mutations associated with resistance (Figure 3B): In most studies with venetoclax-based therapies, the presence of mutations in *FLT3* and *TP53* has been associated with inferior treatment outcomes [94,97,101,102]. Clonal selection of pre-existing, mutated subclones seems to be of major importance for treatment failure and early relapse, as shown in studies with sequential sequencing at baseline and relapse [94,97]. In a study by DiNardo et al., respective samples were investigated for pathogenic mutations conferring resistance. According to the generated data, clonal selection of *FLT3*-ITD-bearing subclones appeared early in treatment, with clinical relapse from one to six months after therapy initiation. In the same study, relapse was driven by emergence of newly diagnosed *FLT3*-ITD mutations in two patients. Interestingly, resistance to venetoclax was also mediated by polyclonal resistance mechanisms, as demonstrated through single-cell sequencing. Here, resistance was due to a series of independent clones that harbored individual mutations in *FLT3* and *NRAS*. In the same study, clonal selection of mutations in *TP53* contributed to one-third of relapses, and clonal outgrowth of *TP53* was observed in all previously mutated cases. In line with these findings, primary resistance was associated with mutations in activating kinases (mainly *FLT3*, *NRAS*/*KRAS*), *TP53*, and *RUNX1* [97]. Similarly, Chyla et al. describe clonal outgrowth of clones with *FLT3*-ITD or *PTPN11* mutation at baseline. Likewise, these mutations were associated with primary resistance to venetoclax in this study [94]. 

#### 4.2.2. Other Mechanisms of Resistance to Venetoclax

Monocytic differentiation (Figure 3C): Interestingly, sensitivity and resistance patterns to venetoclax correlate with level of differentiation in AML [104,105,106]. While the early maturation stages according to the French–American–British (FAB) classification show favorable responses to BCL2 inhibition, monocytic (FAB M5) AML is less sensitive [104,106]. Using gene expression analysis, Bisaillon et al. compared gene expression data in 38 primary AML samples from the Leucegene cohort. While sensitive samples had gene expression signatures associated with hematopoietic stem cells, the gene expression patterns of resistant samples revealed monocytic and inflammatory signatures. Interestingly, AML M1 samples exhibited high levels of BCL2. In contrast, AML M5 samples showed overexpression of antiapoptotic BCL2A1 and MCL1. This finding suggests that different maturation stages rely on different antiapoptotic proteins and potentially explains the different patterns of sensitivity and resistance among the maturation stages of AML [104]. In accordance with these findings, relapses after venetoclax therapy are often of the monocytic subtype, suggesting the selection of clones with alternative antiapoptotic mechanisms under BCL2 inhibition [106]. Similarly, Zhang et al. found BCL2A1 to be upregulated in the M4 and M5 subtypes of AML, which were also the subtypes with the least sensitivity toward venetoclax [105]. Another study showed that in normal hematopoiesis, as well as in the malignant counterparts, expression of BCL2 declined with maturation, while expression of MCL1 increased [106]. When adding the MCL1 inhibitor AZD5991 to venetoclax in an in vitro setting, the authors could observe synthetic lethality in the treated AML cells, despite their initial resistance toward venetoclax [105]. Inhibition of MCL1 or BCLXL was similarly toxic to venetoclax-resistant AML cell lines when given in combination with venetoclax [107]. Clinical research on MCL1 inhibitors is in the early stage, and potential cardiac interactions are currently being investigated [108]. 

Energy metabolism (Figure 3D): In addition to interfering with antiapoptotic mechanisms, the combination of venetoclax with azacytidine was also shown to disrupt cellular energy metabolism by interfering with the tricarboxylic (citric acid) cycle [109]. Leukemia stem cells are particularly reliant on amino acid intake for oxidative phosphorylation (OXPHOS). The combination of venetoclax and azacytidine was shown to interfere with this process, leading to fatal disruption of cellular energy metabolism [110]. Interestingly, the utilization of fatty acids for OXPHOS (fatty acid oxidation) has been identified as a mechanism of resistance to venetoclax/azacytidine. Switching from amino acid oxidation to fatty acid oxidation can occur due to mutations in the RAS pathway or via compensatory adaptation in relapsed disease [111]. Similarly, increased production of NADP+ was described as an alternative source of energy and a potential mechanism of venetoclax/azacytidine resistance [112].

### 4.3. Overcoming Resistance to BCL2 Inhibition

There are various approaches to overcoming resistance to BCL2 inhibition, comprising various stages of preclinical and clinical testing. In the preclinical setting, inhibitors of BCLXL or MCL1 showed strong synergistic killing when combined with venetoclax in resistant AML cell line models with elevated levels of these alternative proapoptotic proteins [107]. S64315, an inhibitor of MCL1, is currently in early clinical testing (NCT03672695, NCT02979366, NCT04629443). Another interesting preclinical approach to overcoming resistance of venetoclax/azacytidine combination therapy is the pharmacologic inhibition of various processes involved in the energy metabolism of leukemic cells [111,112]. 

On the clinical side, the combination of venetoclax with other specific targeted therapies is currently being tested with the aim to achieve longer remissions and to prevent secondary resistance. As discussed, *IDH*-mutated AML favorably responds to venetoclax-based therapy [79,91]. To further improve clinical outcomes, combinations of IDH inhibitors with venetoclax/azacytidine are under investigation. Results from an early clinical trial showed high rates of response and minimal residual disease (MRD) negativity [113]. Other clinical trials investigating venetoclax in combination with ivosidenib or enasidenib are currently ongoing (NCT03471260, NCT04092179). Combining FLT3 inhibitors with a venetoclax-based regimen is another promising therapeutic approach. The combination of midostaurin with decitabine and venetoclax yielded promising results in newly diagnosed elderly *FLT3*-mutated patients with AML with a 2-year OS of 80% [114]. Similarly, the combination of venetoclax with gilteritinib led to high mCRc rates of 75% in r/r *FLT3*-mutated patients with AML, irrespective of prior exposure to FLT3 inhibitors [55]. 

## 5. Other Small Molecules for the Treatment of AML

Pharmacological inhibition of FLT3, IDH, and BCL2 are the most advanced therapeutic options involving small-molecule inhibitors in AML. However, new avenues are being explored for the treatment of AML, such as TP53 reactivation, menin inhibition, or E-selectin inhibition. In addition, glasdegib is another approved small molecule targeting hedgehog signaling. Likewise, HMAs should also be mentioned in the context of this review. 

Based on the BRIGHT AML 1003 study, the hedgehog-signaling inhibitor glasdegib has been approved by the FDA and EMA in combination with low-dose cytarabine for patients ineligible for intensive chemotherapy. Resistance to glasdegib is mechanistically not well understood, but the presence of *DNMT3A* mutations negatively impacted OS in patients with secondary AML within the BRIGHT AML 1003 study [115,116].

Azacytidine and decitabine are the HMAs most frequently used, either alone or in combination with various other antileukemic agents. They cause hypomethylation of DNA, and mutations in epigenetic modifiers such as *IDH* [117], *DNMT3A* [118], and *TET2* [119] have been associated with favorable outcome parameters. On the contrary, mutations in *RUNX1* and *SRSF2* were associated with resistance to HMA therapy [120]. Likewise, metabolism of the drugs seems to play a role in resistance, as low expression levels of genes involved in the activation of the prodrug were associated with worse clinical outcome [121]. Interestingly, HMA therapy also affects expression of proteins involved in immune regulatory processes in myelodysplastic syndromes [122]. However, how far this affects response and resistance patterns is yet poorly understood [123]. 

*TP53* is mutated in 5–15% of AML cases and is associated with a poor prognosis in AML and MDS [1]. Eprenetapopt is a first-in-class small-molecule-reactivating TP53. The substance showed promising data in a phase-II study in combination with azacytidine [124] and was also evaluated in a postallogeneic transplant maintenance setting [125]. Data on the development of resistance to eprenetapopt treatment are scarce. 

Menin is a scaffold protein that is essential for AML with KMT2A fusion proteins or mutated *NPM1* [126]. Menin inhibitors can disrupt the KMT2A–menin complex and therefore, the inhibitors revumenib and ziftodenib are currently being tested in clinical phase-I/II trials [127,128]. According to a study by Perner et al. investigating resistance mechanisms from the phase-I revumenib trial, somatic mutations in *MEN1* affecting the revumenib–menin interface could confer acquired resistance to menin inhibition. These findings were also validated in xenograft models. Of note, the study was the first to demonstrate that chromatin-targeting therapeutic drugs could impose sufficient selection pressure to drive the evolution of escape mutants [129]. 

Another interesting target in AML treatment is E-selectin, as leukemic (stem) cells that bind to E-selectin in vascular niches become more resistant to chemotherapy [130]. This process can be interrupted by uproleselan, a novel E-selectin inhibitor [131]. The first clinical data when combining uproleselan with an intensive chemotherapy regimen in r/r patients showed encouraging results [132]. 

## 6. Conclusions

In less than a decade, small molecules have revolutionized the therapeutic landscape of AML, greatly enlarging the treatment portfolio and improving outcomes. Except for the BCL2 inhibitor venetoclax, most small molecules target molecular lesions that occur only in subsets of AML (i.e., specific mutations), making them a substantial part in the therapeutic concept of precision medicine. Promising new agents targeting different pathogenetic processes in leukemic blasts are under development, leaving us with the hope that we might be able to offer targeted therapies to patients with AML subtypes that cannot yet benefit from this therapeutic concept. 

However, euphoria over targeted treatments is often disrupted by primary resistance or relapses that eventually occur in almost all cases. As discussed in detail in this review, leukemic cells possess a multitude of mechanisms to overcome pharmacologic inhibition. These mechanisms comprise various kinds of molecular processes, from nucleus to cell-to-cell interactions, and we expect that many more will be discovered in the future. Irrespective of the specific nature of an escape mechanism, highly effective clonal selection processes and associated rapid evolution of AML leave physicians and researchers with the challenge of how to durably eliminate this dynamic and adaptable disease.

Combining various small molecules to simultaneously target both a specific lesion and important escape mechanisms appears to be a promising approach (e.g., combining FLT3 inhibition with venetoclax/azacytidine) and leaves us with a growing number of therapeutic options. Among the multitude of escape mechanisms, clonal outgrowth of RAS/RTK pathway mutations seems to be a frequent pattern of resistance to targeted therapies in AML, as well as in other myeloid malignancies [133,134]. Combining RAS/MEK inhibitors with other targeted agents is, therefore, an interesting approach [35]. Other attempts could involve the implementation of (serial) comprehensive or even single-cell sequencing analyses in diagnostics to detect and subsequently treat potential drivers of resistance before they become clinically relevant.

In summary, the small molecules currently available provide a first step in the direction of precision medicine for patients with AML. In the future, sophisticated combination regimens and comprehensive molecular testing will hopefully help us to refine personalization strategies for leukemia treatment and, hence, achieve more and longer-lasting responses. 

## References

[1] Papaemmanuil E., Gerstung M., Bullinger L., Gaidzik V.I., Paschka P., Roberts N.D., Potter N.E., Heuser M., Thol F., Bolli N. (2016). Genomic Classification and Prognosis in Acute Myeloid Leukemia. N. Engl. J. Med..

[2] Döhner H., Wei A.H., Appelbaum F.R., Craddock C., DiNardo C.D., Dombret H., Ebert B.L., Fenaux P., Godley L.A., Hasserjian R.P. (2022). Diagnosis and management of AML in adults: 2022 recommendations from an international expert panel on behalf of the ELN. Blood.

[3] Khoury J.D., Solary E., Abla O., Akkari Y., Alaggio R., Apperley J.F., Bejar R., Berti E., Busque L., Chan J.K.C. (2022). The 5th edition of the World Health Organization Classification of Haematolymphoid Tumours: Myeloid and Histiocytic/Dendritic Neoplasms. Leukemia.

[4] Arber D.A., Orazi A., Hasserjian R.P., Borowitz M.J., Calvo K.R., Kvasnicka H.M., Wang S.A., Bagg A., Barbui T., Branford S. (2022). International Consensus Classification of Myeloid Neoplasms and Acute Leukemias: Integrating morphologic, clinical, and genomic data. Blood.

[5] Rai K.R., Holland J.F., Glidewell O.J., Weinberg V., Brunner K., Obrecht J.P., Preisler H.D., Nawabi I.W., Prager D., Carey R.W. (1981). Treatment of acute myelocytic leukemia: A study by cancer and leukemia group B. Blood.

[6] Fernandez H.F., Sun Z., Yao X., Litzow M.R., Luger S.M., Paietta E.M., Racevskis J., Dewald G.W., Ketterling R.P., Bennett J.M. (2009). Anthracycline Dose Intensification in Acute Myeloid Leukemia. N. Engl. J. Med..

[7] Stone R.M., Mandrekar S.J., Sanford B.L., Laumann K., Geyer S., Bloomfield C.D., Thiede C., Prior T.W., Dohner K., Marcucci G. (2017). Midostaurin plus Chemotherapy for Acute Myeloid Leukemia with a FLT3 Mutation. N. Engl. J. Med..

[8] Rosnet O., Buhring H.J., Marchetto S., Rappold I., Lavagna C., Sainty D., Arnoulet C., Chabannon C., Kanz L., Hannum C. (1996). Human FLT3/FLK2 receptor tyrosine kinase is expressed at the surface of normal and malignant hematopoietic cells. Leukemia.

[9] Ley T.J., Miller C., Ding L., Raphael B.J., Mungall A.J., Robertson A., Hoadley K., Triche T.J., Laird P.W., Baty J.D. (2013). Genomic and epigenomic landscapes of adult de novo acute myeloid leukemia. N. Engl. J. Med..

[10] Agnès F., Shamoon B., Dina C., Rosnet O., Birnbaum D., Galibert F. (1994). Genomic structure of the downstream part of the human FLT3 gene: Exon/intron structure conservation among genes encoding receptor tyrosine kinases (RTK) of subclass III. Gene.

[11] Stirewalt D.L., Radich J.P. (2003). The role of FLT3 in haematopoietic malignancies. Nat. Rev. Cancer.

[12] Daver N., Schlenk R.F., Russell N.H., Levis M.J. (2019). Targeting FLT3 mutations in AML: Review of current knowledge and evidence. Leukemia.

[13] Fröhling S., Schlenk R.F., Breitruck J., Benner A., Kreitmeier S., Tobis K., Döhner H., Döhner K. (2002). Prognostic significance of activating FLT3 mutations in younger adults (16 to 60 years) with acute myeloid leukemia and normal cytogenetics: A study of the AML Study Group Ulm. Blood.

[14] Daver N., Cortes J., Ravandi F., Patel K.P., Burger J.A., Konopleva M., Kantarjian H. (2015). Secondary mutations as mediators of resistance to targeted therapy in leukemia. Blood.

[15] Sawyers C.L. (2002). Finding the next Gleevec: FLT3 targeted kinase inhibitor therapy for acute myeloid leukemia. Cancer Cell.

[16] Antar A.I., Otrock Z.K., Jabbour E., Mohty M., Bazarbachi A. (2020). FLT3 inhibitors in acute myeloid leukemia: Ten frequently asked questions. Leukemia.

[17] Burchert A., Bug G., Fritz L.V., Finke J., Stelljes M., Rollig C., Wollmer E., Wasch R., Bornhauser M., Berg T. (2020). Sorafenib Maintenance After Allogeneic Hematopoietic Stem Cell Transplantation for Acute Myeloid Leukemia With FLT3-Internal Tandem Duplication Mutation (SORMAIN). J. Clin. Oncol..

[18] Röllig C., Serve H., Hüttmann A., Noppeney R., Müller-Tidow C., Krug U., Baldus C.D., Brandts C.H., Kunzmann V., Einsele H. (2015). Addition of sorafenib versus placebo to standard therapy in patients aged 60 years or younger with newly diagnosed acute myeloid leukaemia (SORAML): A multicentre, phase 2, randomised controlled trial. Lancet Oncol..

[19] Röllig C., Serve H., Noppeney R., Hanoun M., Krug U., Baldus C.D., Brandts C.H., Kunzmann V., Einsele H., Krämer A. (2021). Sorafenib or placebo in patients with newly diagnosed acute myeloid leukaemia: Long-term follow-up of the randomized controlled SORAML trial. Leukemia.

[20] Larson R.A., Mandrekar S.J., Huebner L.J., Sanford B.L., Laumann K., Geyer S., Bloomfield C.D., Thiede C., Prior T.W., Döhner K. (2021). Midostaurin reduces relapse in FLT3-mutant acute myeloid leukemia: The Alliance CALGB 10603/RATIFY trial. Leukemia.

[21] Cortes J.E., Khaled S., Martinelli G., Perl A.E., Ganguly S., Russell N., Krämer A., Dombret H., Hogge D., Jonas B.A. (2019). Quizartinib versus salvage chemotherapy in relapsed or refractory FLT3-ITD acute myeloid leukaemia (QuANTUM-R): A multicentre, randomised, controlled, open-label, phase 3 trial. Lancet Oncol..

[22] Erba H.P., Montesinos P., Kim H.J., Patkowska E., Vrhovac R., Žák P., Wang P.N., Mitov T., Hanyok J., Kamel Y.M. (2023). Quizartinib plus chemotherapy in newly diagnosed patients with FLT3-internal-tandem-duplication-positive acute myeloid leukaemia (QuANTUM-First): A randomised, double-blind, placebo-controlled, phase 3 trial. Lancet.

[23] Perl A.E., Martinelli G., Cortes J.E., Neubauer A., Berman E., Paolini S., Montesinos P., Baer M.R., Larson R.A., Ustun C. (2019). Gilteritinib or Chemotherapy for Relapsed or Refractory FLT3-Mutated AML. N. Engl. J. Med..

[24] Joshi S.K., Sharzehi S., Pittsenbarger J., Bottomly D., Tognon C.E., McWeeney S.K., Druker B.J., Traer E. (2021). A noncanonical FLT3 gatekeeper mutation disrupts gilteritinib binding and confers resistance. Am. J. Hematol..

[25] Williams A.B., Nguyen B., Li L., Brown P., Levis M., Leahy D., Small D. (2013). Mutations of FLT3/ITD confer resistance to multiple tyrosine kinase inhibitors. Leukemia.

[26] Schmalbrock L.K., Dolnik A., Cocciardi S., Strang E., Theis F., Jahn N., Panina E., Blatte T.J., Herzig J., Skambraks S. (2021). Clonal evolution of acute myeloid leukemia with FLT3-ITD mutation under treatment with midostaurin. Blood.

[27] Sharzehi S., Joshi S.K., Pittsenbarger J., Tyner J.W., Traer E. (2021). The FLT3 F691L Gatekeeper Mutation Promotes Clinical Resistance to Gilteritinib + Venetoclax (GILT + VEN) in AML. Blood.

[28] Smith C.C., Paguirigan A., Jeschke G.R., Lin K.C., Massi E., Tarver T., Chin C.S., Asthana S., Olshen A., Travers K.J. (2017). Heterogeneous resistance to quizartinib in acute myeloid leukemia revealed by single-cell analysis. Blood.

[29] Heidel F., Solem F.K., Breitenbuecher F., Lipka D.B., Kasper S., Thiede M.H., Brandts C., Serve H., Roesel J., Giles F. (2006). Clinical resistance to the kinase inhibitor PKC412 in acute myeloid leukemia by mutation of Asn-676 in the FLT3 tyrosine kinase domain. Blood.

[30] Zhang H., Savage S., Schultz A.R., Bottomly D., White L., Segerdell E., Wilmot B., McWeeney S.K., Eide C.A., Nechiporuk T. (2019). Clinical resistance to crenolanib in acute myeloid leukemia due to diverse molecular mechanisms. Nat. Commun..

[31] Smith C.C., Lin K., Stecula A., Sali A., Shah N.P. (2015). FLT3 D835 mutations confer differential resistance to type II FLT3 inhibitors. Leukemia.

[32] Yilmaz M., Alfayez M., DiNardo C.D., Borthakur G., Kadia T.M., Konopleva M.Y., Loghavi S., Kanagal-Shamanna R., Patel K.P., Jabbour E.J. (2020). Outcomes with sequential FLT3-inhibitor-based therapies in patients with AML. J. Hematol. Oncol..

[33] Lindblad O., Cordero E., Puissant A., Macaulay L., Ramos A., Kabir N.N., Sun J., Vallon-Christersson J., Haraldsson K., Hemann M.T. (2016). Aberrant activation of the PI3K/mTOR pathway promotes resistance to sorafenib in AML. Oncogene.

[34] Alotaibi A.S., Yilmaz M., Kanagal-Shamanna R., Loghavi S., Kadia T.M., DiNardo C.D., Borthakur G., Konopleva M., Pierce S.A., Wang S.A. (2021). Patterns of Resistance Differ in Patients with Acute Myeloid Leukemia Treated with Type I versus Type II FLT3 inhibitors. Blood Cancer Discov..

[35] McMahon C.M., Ferng T., Canaani J., Wang E.S., Morrissette J.J.D., Eastburn D.J., Pellegrino M., Durruthy-Durruthy R., Watt C.D., Asthana S. (2019). Clonal Selection with RAS Pathway Activation Mediates Secondary Clinical Resistance to Selective FLT3 Inhibition in Acute Myeloid Leukemia. Cancer Discov..

[36] Rummelt C., Gorantla S.P., Meggendorfer M., Charlet A., Endres C., Döhner K., Heidel F.H., Fischer T., Haferlach T., Duyster J. (2021). Activating JAK-mutations confer resistance to FLT3 kinase inhibitors in FLT3-ITD positive AML in vitro and in vivo. Leukemia.

[37] Patel R.K., Weir M.C., Shen K., Snyder D., Cooper V.S., Smithgall T.E. (2019). Expression of myeloid Src-family kinases is associated with poor prognosis in AML and influences Flt3-ITD kinase inhibitor acquired resistance. PLoS ONE.

[38] Breitenbuecher F., Markova B., Kasper S., Carius B., Stauder T., Böhmer F.D., Masson K., Rönnstrand L., Huber C., Kindler T. (2009). A novel molecular mechanism of primary resistance to FLT3-kinase inhibitors in AML. Blood.

[39] Kohl T.M., Hellinger C., Ahmed F., Buske C., Hiddemann W., Bohlander S.K., Spiekermann K. (2007). BH3 mimetic ABT-737 neutralizes resistance to FLT3 inhibitor treatment mediated by FLT3-independent expression of BCL2 in primary AML blasts. Leukemia.

[40] Hunter H.M., Pallis M., Seedhouse C.H., Grundy M., Gray C., Russell N.H. (2004). The expression of P-glycoprotein in AML cells with FLT3 internal tandem duplications is associated with reduced apoptosis in response to FLT3 inhibitors. Br. J. Haematol..

[41] Eguchi M., Minami Y., Kuzume A., Chi S. (2020). Mechanisms Underlying Resistance to FLT3 Inhibitors in Acute Myeloid Leukemia. Biomedicines.

[42] Kumar B., Garcia M., Weng L., Jung X., Murakami J.L., Hu X., McDonald T., Lin A., Kumar A.R., DiGiusto D.L. (2018). Acute myeloid leukemia transforms the bone marrow niche into a leukemia-permissive microenvironment through exosome secretion. Leukemia.

[43] Sato T., Yang X., Knapper S., White P., Smith B.D., Galkin S., Small D., Burnett A., Levis M. (2011). FLT3 ligand impedes the efficacy of FLT3 inhibitors in vitro and in vivo. Blood.

[44] Chang Y.T., Hernandez D., Alonso S., Gao M., Su M., Ghiaur G., Levis M.J., Jones R.J. (2019). Role of CYP3A4 in bone marrow microenvironment-mediated protection of FLT3/ITD AML from tyrosine kinase inhibitors. Blood Adv..

[45] Park I.K., Mundy-Bosse B., Whitman S.P., Zhang X., Warner S.L., Bearss D.J., Blum W., Marcucci G., Caligiuri M.A. (2015). Receptor tyrosine kinase Axl is required for resistance of leukemic cells to FLT3-targeted therapy in acute myeloid leukemia. Leukemia.

[46] Dumas P.Y., Naudin C., Martin-Lannerée S., Izac B., Casetti L., Mansier O., Rousseau B., Artus A., Dufossée M., Giese A. (2019). Hematopoietic niche drives FLT3-ITD acute myeloid leukemia resistance to quizartinib via STAT5-and hypoxia-dependent upregulation of AXL. Haematologica.

[47] Young D.J., Nguyen B., Li L., Higashimoto T., Levis M.J., Liu J.O., Small D. (2021). A Method for Overcoming Plasma Protein Inhibition of Tyrosine Kinase Inhibitors. Blood Cancer Discov..

[48] Zhang Y., Wang P., Wang Y., Shen Y. (2023). Sitravatinib as a potent FLT3 inhibitor can overcome gilteritinib resistance in acute myeloid leukemia. Biomark. Res..

[49] Yamaura T., Nakatani T., Uda K., Ogura H., Shin W., Kurokawa N., Saito K., Fujikawa N., Date T., Takasaki M. (2018). A novel irreversible FLT3 inhibitor, FF-10101, shows excellent efficacy against AML cells with FLT3 mutations. Blood.

[50] Levis M.J., Smith C.C., Perl A.E., Schiller G.J., Fathi A.T., Roboz G.J., Wang E.S., Altman J.K., Ando M., Suzuki T. (2021). Phase 1 first-in-human study of irreversible FLT3 inhibitor FF-10101-01 in relapsed or refractory acute myeloid leukemia. J. Clin. Oncol..

[51] Singh Mali R., Zhang Q., DeFilippis R.A., Cavazos A., Kuruvilla V.M., Raman J., Mody V., Choo E.F., Dail M., Shah N.P. (2021). Venetoclax combines synergistically with FLT3 inhibition to effectively target leukemic cells in FLT3-ITD+ acute myeloid leukemia models. Haematologica.

[52] Brinton L.T., Zhang P., Williams K., Canfield D., Orwick S., Sher S., Wasmuth R., Beaver L., Cempre C., Skinner J. (2020). Synergistic effect of BCL2 and FLT3 co-inhibition in acute myeloid leukemia. J. Hematol. Oncol..

[53] Ma J., Zhao S., Qiao X., Knight T., Edwards H., Polin L., Kushner J., Dzinic S.H., White K., Wang G. (2019). Inhibition of Bcl-2 Synergistically Enhances the Antileukemic Activity of Midostaurin and Gilteritinib in Preclinical Models of FLT3-Mutated Acute Myeloid Leukemia. Clin. Cancer Res..

[54] Zhu R., Li L., Nguyen B., Seo J., Wu M., Seale T., Levis M., Duffield A., Hu Y., Small D. (2021). FLT3 tyrosine kinase inhibitors synergize with BCL-2 inhibition to eliminate FLT3/ITD acute leukemia cells through BIM activation. Signal Transduct. Target. Ther..

[55] Daver N., Perl A.E., Maly J., Levis M., Ritchie E., Litzow M., McCloskey J., Smith C.C., Schiller G., Bradley T. (2022). Venetoclax Plus Gilteritinib for FLT3-Mutated Relapsed/Refractory Acute Myeloid Leukemia. J. Clin. Oncol..

[56] Janssen M., Schmidt C., Bruch P.M., Blank M.F., Rohde C., Waclawiczek A., Heid D., Renders S., Göllner S., Vierbaum L. (2022). Venetoclax synergizes with gilteritinib in FLT3 wild-type high-risk acute myeloid leukemia by suppressing MCL-1. Blood.

[57] Weisberg E., Liu Q., Nelson E., Kung A.L., Christie A.L., Bronson R., Sattler M., Sanda T., Zhao Z., Hur W. (2012). Using combination therapy to override stromal-mediated chemoresistance in mutant FLT3-positive AML: Synergism between FLT3 inhibitors, dasatinib/multi-targeted inhibitors and JAK inhibitors. Leukemia.

[58] Weisberg E., Liu Q., Zhang X., Nelson E., Sattler M., Liu F., Nicolais M., Zhang J., Mitsiades C., Smith R.W. (2013). Selective Akt inhibitors synergize with tyrosine kinase inhibitors and effectively override stroma-associated cytoprotection of mutant FLT3-positive AML cells. PLoS ONE.

[59] Kapoor S., Natarajan K., Baldwin P.R., Doshi K.A., Lapidus R.G., Mathias T.J., Scarpa M., Trotta R., Davila E., Kraus M. (2018). Concurrent Inhibition of Pim and FLT3 Kinases Enhances Apoptosis of FLT3-ITD Acute Myeloid Leukemia Cells through Increased Mcl-1 Proteasomal Degradation. Clin. Cancer Res..

[60] Traer E., Martinez J., Javidi-Sharifi N., Agarwal A., Dunlap J., English I., Kovacsovics T., Tyner J.W., Wong M., Druker B.J. (2016). FGF2 from Marrow Microenvironment Promotes Resistance to FLT3 Inhibitors in Acute Myeloid Leukemia. Cancer Res..

[61] Zeng Z., Shi Y.X., Samudio I.J., Wang R.Y., Ling X., Frolova O., Levis M., Rubin J.B., Negrin R.R., Estey E.H. (2009). Targeting the leukemia microenvironment by CXCR4 inhibition overcomes resistance to kinase inhibitors and chemotherapy in AML. Blood.

[62] Zhang W., Yu G., Zhang H., Basyal M., Ly C., Yuan B., Ruvolo V., Piya S., Bhattacharya S., Zhang Q. (2023). Concomitant targeting of FLT3 and BTK overcomes FLT3 inhibitor resistance in acute myeloid leukemia through the inhibition of autophagy. Haematologica.

[63] Marcucci G., Maharry K., Wu Y.Z., Radmacher M.D., Mrózek K., Margeson D., Holland K.B., Whitman S.P., Becker H., Schwind S. (2010). IDH1 and IDH2 gene mutations identify novel molecular subsets within de novo cytogenetically normal acute myeloid leukemia: A Cancer and Leukemia Group B study. J. Clin. Oncol..

[64] Mardis E.R., Ding L., Dooling D.J., Larson D.E., McLellan M.D., Chen K., Koboldt D.C., Fulton R.S., Delehaunty K.D., McGrath S.D. (2009). Recurring mutations found by sequencing an acute myeloid leukemia genome. N. Engl. J. Med..

[65] Wagner K., Damm F., Göhring G., Görlich K., Heuser M., Schäfer I., Ottmann O., Lübbert M., Heit W., Kanz L. (2010). Impact of IDH1 R132 mutations and an IDH1 single nucleotide polymorphism in cytogenetically normal acute myeloid leukemia: SNP rs11554137 is an adverse prognostic factor. J. Clin. Oncol..

[66] Dang L., White D.W., Gross S., Bennett B.D., Bittinger M.A., Driggers E.M., Fantin V.R., Jang H.G., Jin S., Keenan M.C. (2009). Cancer-associated IDH1 mutations produce 2-hydroxyglutarate. Nature.

[67] Xu W., Yang H., Liu Y., Yang Y., Wang P., Kim S.H., Ito S., Yang C., Wang P., Xiao M.T. (2011). Oncometabolite 2-hydroxyglutarate is a competitive inhibitor of alpha-ketoglutarate-dependent dioxygenases. Cancer Cell.

[68] Figueroa M.E., Abdel-Wahab O., Lu C., Ward P.S., Patel J., Shih A., Li Y., Bhagwat N., Vasanthakumar A., Fernandez H.F. (2010). Leukemic IDH1 and IDH2 mutations result in a hypermethylation phenotype, disrupt TET2 function, and impair hematopoietic differentiation. Cancer Cell.

[69] Lu C., Ward P.S., Kapoor G.S., Rohle D., Turcan S., Abdel-Wahab O., Edwards C.R., Khanin R., Figueroa M.E., Melnick A. (2012). IDH mutation impairs histone demethylation and results in a block to cell differentiation. Nature.

[70] Popovici-Muller J., Lemieux R.M., Artin E., Saunders J.O., Salituro F.G., Travins J., Cianchetta G., Cai Z., Zhou D., Cui D. (2018). Discovery of AG-120 (Ivosidenib): A First-in-Class Mutant IDH1 Inhibitor for the Treatment of IDH1 Mutant Cancers. ACS Med. Chem. Lett..

[71] Yen K., Travins J., Wang F., David M.D., Artin E., Straley K., Padyana A., Gross S., DeLaBarre B., Tobin E. (2017). AG-221, a First-in-Class Therapy Targeting Acute Myeloid Leukemia Harboring Oncogenic IDH2 Mutations. Cancer Discov..

[72] Roboz G.J., DiNardo C.D., Stein E.M., de Botton S., Mims A.S., Prince G.T., Altman J.K., Arellano M.L., Donnellan W., Erba H.P. (2020). Ivosidenib induces deep durable remissions in patients with newly diagnosed IDH1-mutant acute myeloid leukemia. Blood.

[73] DiNardo C.D., Stein E.M., de Botton S., Roboz G.J., Altman J.K., Mims A.S., Swords R., Collins R.H., Mannis G.N., Pollyea D.A. (2018). Durable Remissions with Ivosidenib in IDH1-Mutated Relapsed or Refractory AML. N. Engl. J. Med..

[74] Montesinos P., Recher C., Vives S., Zarzycka E., Wang J., Bertani G., Heuser M., Calado R.T., Schuh A.C., Yeh S.P. (2022). Ivosidenib and Azacitidine in IDH1-Mutated Acute Myeloid Leukemia. N. Engl. J. Med..

[75] De Botton S., Fenaux P., Yee K., Récher C., Wei A.H., Montesinos P., Taussig D.C., Pigneux A., Braun T., Curti A. (2023). Olutasidenib (FT-2102) induces durable complete remissions in patients with relapsed or refractory IDH1-mutated AML. Blood Adv..

[76] Watts J.M., Baer M.R., Yang J., Prebet T., Lee S., Schiller G.J., Dinner S.N., Pigneux A., Montesinos P., Wang E.S. (2023). Olutasidenib alone or with azacitidine in IDH1-mutated acute myeloid leukaemia and myelodysplastic syndrome: Phase 1 results of a phase 1/2 trial. Lancet Haematol..

[77] Stein E.M., DiNardo C.D., Pollyea D.A., Fathi A.T., Roboz G.J., Altman J.K., Stone R.M., DeAngelo D.J., Levine R.L., Flinn I.W. (2017). Enasidenib in mutant IDH2 relapsed or refractory acute myeloid leukemia. Blood.

[78] Pollyea D.A., Tallman M.S., de Botton S., Kantarjian H.M., Collins R., Stein A.S., Frattini M.G., Xu Q., Tosolini A., See W.L. (2019). Enasidenib, an inhibitor of mutant IDH2 proteins, induces durable remissions in older patients with newly diagnosed acute myeloid leukemia. Leukemia.

[79] DiNardo C.D., Schuh A.C., Stein E.M., Montesinos P., Wei A.H., de Botton S., Zeidan A.M., Fathi A.T., Kantarjian H.M., Bennett J.M. (2021). Enasidenib plus azacitidine versus azacitidine alone in patients with newly diagnosed, mutant-IDH2 acute myeloid leukaemia (AG221-AML-005): A single-arm, phase 1b and randomised, phase 2 trial. Lancet Oncol..

[80] Choe S., Wang H., DiNardo C.D., Stein E.M., de Botton S., Roboz G.J., Altman J.K., Mims A.S., Watts J.M., Pollyea D.A. (2020). Molecular mechanisms mediating relapse following ivosidenib monotherapy in IDH1-mutant relapsed or refractory AML. Blood Adv..

[81] Amatangelo M.D., Quek L., Shih A., Stein E.M., Roshal M., David M.D., Marteyn B., Farnoud N.R., de Botton S., Bernard O.A. (2017). Enasidenib induces acute myeloid leukemia cell differentiation to promote clinical response. Blood.

[82] Intlekofer A.M., Shih A.H., Wang B., Nazir A., Rustenburg A.S., Albanese S.K., Patel M., Famulare C., Correa F.M., Takemoto N. (2018). Acquired resistance to IDH inhibition through trans or cis dimer-interface mutations. Nature.

[83] Harding J.J., Lowery M.A., Shih A.H., Schvartzman J.M., Hou S., Famulare C., Patel M., Roshal M., Do R.K., Zehir A. (2018). Isoform Switching as a Mechanism of Acquired Resistance to Mutant Isocitrate Dehydrogenase Inhibition. Cancer Discov..

[84] Wang F., Morita K., DiNardo C.D., Furudate K., Tanaka T., Yan Y., Patel K.P., MacBeth K.J., Wu B., Liu G. (2021). Leukemia stemness and co-occurring mutations drive resistance to IDH inhibitors in acute myeloid leukemia. Nat. Commun..

[85] Stein E.M., DiNardo C.D., Fathi A.T., Pollyea D.A., Stone R.M., Altman J.K., Roboz G.J., Patel M.R., Collins R., Flinn I.W. (2019). Molecular remission and response patterns in patients with mutant-IDH2 acute myeloid leukemia treated with enasidenib. Blood.

[86] Quek L., David M.D., Kennedy A., Metzner M., Amatangelo M., Shih A., Stoilova B., Quivoron C., Heiblig M., Willekens C. (2018). Clonal heterogeneity of acute myeloid leukemia treated with the IDH2 inhibitor enasidenib. Nat. Med..

[87] Youle R.J., Strasser A. (2008). The BCL-2 protein family: Opposing activities that mediate cell death. Nat. Rev. Mol. Cell Biol..

[88] Campos L., Rouault J.P., Sabido O., Oriol P., Roubi N., Vasselon C., Archimbaud E., Magaud J.P., Guyotat D. (1993). High expression of bcl-2 protein in acute myeloid leukemia cells is associated with poor response to chemotherapy. Blood.

[89] Bensi L., Longo R., Vecchi A., Messora C., Garagnani L., Bernardi S., Tamassia M.G., Sacchi S. (1995). Bcl-2 oncoprotein expression in acute myeloid leukemia. Haematologica.

[90] Konopleva M., Pollyea D.A., Potluri J., Chyla B., Hogdal L., Busman T., McKeegan E., Salem A.H., Zhu M., Ricker J.L. (2016). Efficacy and Biological Correlates of Response in a Phase II Study of Venetoclax Monotherapy in Patients with Acute Myelogenous Leukemia. Cancer Discov..

[91] DiNardo C.D., Jonas B.A., Pullarkat V., Thirman M.J., Garcia J.S., Wei A.H., Konopleva M., Dohner H., Letai A., Fenaux P. (2020). Azacitidine and Venetoclax in Previously Untreated Acute Myeloid Leukemia. N. Engl. J. Med..

[92] Wei A.H., Montesinos P., Ivanov V., DiNardo C.D., Novak J., Laribi K., Kim I., Stevens D.A., Fiedler W., Pagoni M. (2020). Venetoclax plus LDAC for newly diagnosed AML ineligible for intensive chemotherapy: A phase 3 randomized placebo-controlled trial. Blood.

[93] Jin S., Cojocari D., Purkal J.J., Popovic R., Talaty N.N., Xiao Y., Solomon L.R., Boghaert E.R., Leverson J.D., Phillips D.C. (2020). 5-Azacitidine Induces NOXA to Prime AML Cells for Venetoclax-Mediated Apoptosis. Clin. Cancer Res..

[94] Chyla B., Daver N., Doyle K., McKeegan E., Huang X., Ruvolo V., Wang Z., Chen K., Souers A., Leverson J. (2018). Genetic Biomarkers Of Sensitivity and Resistance to Venetoclax Monotherapy in Patients With Relapsed Acute Myeloid Leukemia. Am. J. Hematol..

[95] Pollyea D.A., Pratz K., Letai A., Jonas B.A., Wei A.H., Pullarkat V., Konopleva M., Thirman M.J., Arellano M., Becker P.S. (2021). Venetoclax with azacitidine or decitabine in patients with newly diagnosed acute myeloid leukemia: Long term follow-up from a phase 1b study. Am. J. Hematol..

[96] Pollyea D.A., DiNardo C.D., Arellano M.L., Pigneux A., Fiedler W., Konopleva M., Rizzieri D.A., Smith B.D., Shinagawa A., Lemoli R.M. (2022). Impact of Venetoclax and Azacitidine in Treatment-Naïve Patients with Acute Myeloid Leukemia and IDH1/2 Mutations. Clin. Cancer Res..

[97] DiNardo C.D., Tiong I.S., Quaglieri A., MacRaild S., Loghavi S., Brown F.C., Thijssen R., Pomilio G., Ivey A., Salmon J.M. (2020). Molecular patterns of response and treatment failure after frontline venetoclax combinations in older patients with AML. Blood.

[98] Chan S.M., Thomas D., Corces-Zimmerman M.R., Xavy S., Rastogi S., Hong W.J., Zhao F., Medeiros B.C., Tyvoll D.A., Majeti R. (2015). Isocitrate dehydrogenase 1 and 2 mutations induce BCL-2 dependence in acute myeloid leukemia. Nat. Med..

[99] Aldoss I., Yang D., Pillai R., Sanchez J.F., Mei M., Aribi A., Ali H., Sandhu K., Al Malki M.M., Salhotra A. (2019). Association of leukemia genetics with response to venetoclax and hypomethylating agents in relapsed/refractory acute myeloid leukemia. Am. J. Hematol..

[100] DiNardo C.D., Pratz K., Pullarkat V., Jonas B.A., Arellano M., Becker P.S., Frankfurt O., Konopleva M., Wei A.H., Kantarjian H.M. (2019). Venetoclax combined with decitabine or azacitidine in treatment-naive, elderly patients with acute myeloid leukemia. Blood.

[101] Wang Y.W., Tsai C.H., Lin C.C., Tien F.M., Chen Y.W., Lin H.Y., Yao M., Lin Y.C., Lin C.T., Cheng C.L. (2020). Cytogenetics and mutations could predict outcome in relapsed and refractory acute myeloid leukemia patients receiving BCL-2 inhibitor venetoclax. Ann. Hematol..

[102] Chua C.C., Roberts A.W., Reynolds J., Fong C.Y., Ting S.B., Salmon J.M., MacRaild S., Ivey A., Tiong I.S., Fleming S. (2020). Chemotherapy and Venetoclax in Elderly Acute Myeloid Leukemia Trial (CAVEAT): A Phase Ib Dose-Escalation Study of Venetoclax Combined With Modified Intensive Chemotherapy. J. Clin. Oncol..

[103] Griffioen M.S., de Leeuw D.C., Janssen J., Smit L. (2022). Targeting Acute Myeloid Leukemia with Venetoclax; Biomarkers for Sensitivity and Rationale for Venetoclax-Based Combination Therapies. Cancers.

[104] Bisaillon R., Moison C., Thiollier C., Krosl J., Bordeleau M.E., Lehnertz B., Lavallee V.P., MacRae T., Mayotte N., Labelle C. (2020). Genetic characterization of ABT-199 sensitivity in human AML. Leukemia.

[105] Zhang H., Nakauchi Y., Kohnke T., Stafford M., Bottomly D., Thomas R., Wilmot B., McWeeney S.K., Majeti R., Tyner J.W. (2020). Integrated analysis of patient samples identifies biomarkers for venetoclax efficacy and combination strategies in acute myeloid leukemia. Nat. Cancer.

[106] Pei S., Pollyea D.A., Gustafson A., Stevens B.M., Minhajuddin M., Fu R., Riemondy K.A., Gillen A.E., Sheridan R.M., Kim J. (2020). Monocytic Subclones Confer Resistance to Venetoclax-Based Therapy in Patients with Acute Myeloid Leukemia. Cancer Discov..

[107] Tahir S.K., Smith M.L., Hessler P., Rapp L.R., Idler K.B., Park C.H., Leverson J.D., Lam L.T. (2017). Potential mechanisms of resistance to venetoclax and strategies to circumvent it. BMC Cancer.

[108] Tantawy S.I., Sarkar A., Hubner S., Tan Z., Wierda W.G., Eldeib A., Zhang S., Kornblau S., Gandhi V. (2023). Mechanisms of MCL-1 Protein Stability Induced by MCL-1 Antagonists in B-Cell Malignancies. Clin. Cancer Res..

[109] Pollyea D.A., Stevens B.M., Jones C.L., Winters A., Pei S., Minhajuddin M., D’Alessandro A., Culp-Hill R., Riemondy K.A., Gillen A.E. (2018). Venetoclax with azacitidine disrupts energy metabolism and targets leukemia stem cells in patients with acute myeloid leukemia. Nat. Med..

[110] Jones C.L., Stevens B.M., D’Alessandro A., Reisz J.A., Culp-Hill R., Nemkov T., Pei S., Khan N., Adane B., Ye H. (2018). Inhibition of Amino Acid Metabolism Selectively Targets Human Leukemia Stem Cells. Cancer Cell.

[111] Stevens B.M., Jones C.L., Pollyea D.A., Culp-Hill R., D’Alessandro A., Winters A., Krug A., Abbott D., Goosman M., Pei S. (2020). Fatty acid metabolism underlies venetoclax resistance in acute myeloid leukemia stem cells. Nat. Cancer.

[112] Jones C.L., Stevens B.M., Pollyea D.A., Culp-Hill R., Reisz J.A., Nemkov T., Gehrke S., Gamboni F., Krug A., Winters A. (2020). Nicotinamide Metabolism Mediates Resistance to Venetoclax in Relapsed Acute Myeloid Leukemia Stem Cells. Cell Stem Cell.

[113] Lachowiez C.A., Loghavi S., Zeng Z., Tanaka T., Kim Y.J., Uryu H., Turkalj S., Jakobsen N.A., Luskin M.R., Duose D.Y. (2023). A Phase Ib/II Study of Ivosidenib with Venetoclax ± Azacitidine in IDH1-Mutated Myeloid Malignancies. Blood Cancer Discov..

[114] Maiti A., DiNardo C.D., Daver N.G., Rausch C.R., Ravandi F., Kadia T.M., Pemmaraju N., Borthakur G., Bose P., Issa G.C. (2021). Triplet therapy with venetoclax, FLT3 inhibitor and decitabine for FLT3-mutated acute myeloid leukemia. Blood Cancer J..

[115] Cortes J.E., Heidel F.H., Hellmann A., Fiedler W., Smith B.D., Robak T., Montesinos P., Pollyea D.A., DesJardins P., Ottmann O. (2019). Randomized comparison of low dose cytarabine with or without glasdegib in patients with newly diagnosed acute myeloid leukemia or high-risk myelodysplastic syndrome. Leukemia.

[116] Heuser M., Smith B.D., Fiedler W., Sekeres M.A., Montesinos P., Leber B., Merchant A., Papayannidis C., Pérez-Simón J.A., Hoang C.J. (2021). Clinical benefit of glasdegib plus low-dose cytarabine in patients with de novo and secondary acute myeloid leukemia: Long-term analysis of a phase II randomized trial. Ann. Hematol..

[117] Emadi A., Faramand R., Carter-Cooper B., Tolu S., Ford L.A., Lapidus R.G., Wetzler M., Wang E.S., Etemadi A., Griffiths E.A. (2015). Presence of isocitrate dehydrogenase mutations may predict clinical response to hypomethylating agents in patients with acute myeloid leukemia. Am. J. Hematol..

[118] Metzeler K.H., Walker A., Geyer S., Garzon R., Klisovic R.B., Bloomfield C.D., Blum W., Marcucci G. (2012). DNMT3A mutations and response to the hypomethylating agent decitabine in acute myeloid leukemia. Leukemia.

[119] Itzykson R., Kosmider O., Cluzeau T., Mansat-De Mas V., Dreyfus F., Beyne-Rauzy O., Quesnel B., Vey N., Gelsi-Boyer V., Raynaud S. (2011). Impact of TET2 mutations on response rate to azacitidine in myelodysplastic syndromes and low blast count acute myeloid leukemias. Leukemia.

[120] Welch J.S., Petti A.A., Miller C.A., Fronick C.C., O’Laughlin M., Fulton R.S., Wilson R.K., Baty J.D., Duncavage E.J., Tandon B. (2016). TP53 and Decitabine in Acute Myeloid Leukemia and Myelodysplastic Syndromes. N. Engl. J. Med..

[121] Valencia A., Masala E., Rossi A., Martino A., Sanna A., Buchi F., Canzian F., Cilloni D., Gaidano V., Voso M.T. (2014). Expression of nucleoside-metabolizing enzymes in myelodysplastic syndromes and modulation of response to azacitidine. Leukemia.

[122] Yang H., Bueso-Ramos C., DiNardo C., Estecio M.R., Davanlou M., Geng Q.R., Fang Z., Nguyen M., Pierce S., Wei Y. (2014). Expression of PD-L1, PD-L2, PD-1 and CTLA4 in myelodysplastic syndromes is enhanced by treatment with hypomethylating agents. Leukemia.

[123] Zhao G., Wang Q., Li S., Wang X. (2021). Resistance to Hypomethylating Agents in Myelodysplastic Syndrome and Acute Myeloid Leukemia From Clinical Data and Molecular Mechanism. Front. Oncol..

[124] Cluzeau T., Sebert M., Rahme R., Cuzzubbo S., Lehmann-Che J., Madelaine I., Peterlin P., Beve B., Attalah H., Chermat F. (2021). Eprenetapopt Plus Azacitidine in TP53-Mutated Myelodysplastic Syndromes and Acute Myeloid Leukemia: A Phase II Study by the Groupe Francophone des Myelodysplasies (GFM). J. Clin. Oncol..

[125] Mishra A., Tamari R., DeZern A.E., Byrne M.T., Gooptu M., Chen Y.B., Deeg H.J., Sallman D., Gallacher P., Wennborg A. (2022). Eprenetapopt Plus Azacitidine After Allogeneic Hematopoietic Stem-Cell Transplantation for TP53-Mutant Acute Myeloid Leukemia and Myelodysplastic Syndromes. J. Clin. Oncol..

[126] Issa G.C., Ravandi F., DiNardo C.D., Jabbour E., Kantarjian H.M., Andreeff M. (2021). Therapeutic implications of menin inhibition in acute leukemias. Leukemia.

[127] Issa G.C., Aldoss I., DiPersio J., Cuglievan B., Stone R., Arellano M., Thirman M.J., Patel M.R., Dickens D.S., Shenoy S. (2023). The menin inhibitor revumenib in KMT2A-rearranged or NPM1-mutant leukaemia. Nature.

[128] Erba H.P., Fathi A.T., Issa G.C., Altman J.K., Montesinos P., Patnaik M.M., Foran J.M., De Botton S., Baer M.R., Schiller G.J. (2022). Update on a Phase 1/2 First-in-Human Study of the Menin-KMT2A (MLL) Inhibitor Ziftomenib (KO-539) in Patients with Relapsed or Refractory Acute Myeloid Leukemia. Blood.

[129] Perner F., Stein E.M., Wenge D.V., Singh S., Kim J., Apazidis A., Rahnamoun H., Anand D., Marinaccio C., Hatton C. (2023). MEN1 mutations mediate clinical resistance to menin inhibition. Nature.

[130] Erbani J., Tay J., Barbier V., Levesque J.P., Winkler I.G. (2020). Acute Myeloid Leukemia Chemo-Resistance Is Mediated by E-selectin Receptor CD162 in Bone Marrow Niches. Front. Cell Dev. Biol..

[131] Barbier V., Erbani J., Fiveash C., Davies J.M., Tay J., Tallack M.R., Lowe J., Magnani J.L., Pattabiraman D.R., Perkins A.C. (2020). Endothelial E-selectin inhibition improves acute myeloid leukaemia therapy by disrupting vascular niche-mediated chemoresistance. Nat. Commun..

[132] DeAngelo D.J., Jonas B.A., Liesveld J.L., Bixby D.L., Advani A.S., Marlton P., Magnani J.L., Thackray H.M., Feldman E.J., O’Dwyer M.E. (2022). Phase 1/2 study of uproleselan added to chemotherapy in patients with relapsed or refractory acute myeloid leukemia. Blood.

[133] Mylonas E., Yoshida K., Frick M., Hoyer K., Christen F., Kaeda J., Obenaus M., Noerenberg D., Hennch C., Chan W. (2020). Single-cell analysis based dissection of clonality in myelofibrosis. Nat. Commun..

[134] Suzuki M., Abe A., Imagama S., Nomura Y., Tanizaki R., Minami Y., Hayakawa F., Ito Y., Katsumi A., Yamamoto K. (2010). BCR-ABL-independent and RAS / MAPK pathway-dependent form of imatinib resistance in Ph-positive acute lymphoblastic leukemia cell line with activation of EphB4. Eur. J. Haematol..

